# Comparison of evolutionary algorithms in gene regulatory network model inference

**DOI:** 10.1186/1471-2105-11-59

**Published:** 2010-01-27

**Authors:** Alina Sîrbu, Heather J Ruskin, Martin Crane

**Affiliations:** 1 Centre for Scientific Computing and Complex Systems Modelling, Dublin City University, Dublin 9, Ireland

## Abstract

**Background:**

The evolution of high throughput technologies that measure gene expression levels has created a data base for inferring GRNs (a process also known as *reverse engineering *of GRNs). However, the nature of these data has made this process very difficult. At the moment, several methods of discovering *qualitative *causal relationships between genes with high accuracy from microarray data exist, but large scale *quantitative *analysis on real biological datasets cannot be performed, to date, as existing approaches are not suitable for real microarray data which are noisy and insufficient.

**Results:**

This paper performs an analysis of several existing evolutionary algorithms for *quantitative *gene regulatory network modelling. The aim is to present the techniques used and offer a comprehensive comparison of approaches, under a common framework. Algorithms are applied to both synthetic and real gene expression data from DNA microarrays, and ability to reproduce biological behaviour, scalability and robustness to noise are assessed and compared.

**Conclusions:**

Presented is a comparison framework for assessment of evolutionary algorithms, used to infer gene regulatory networks. Promising methods are identified and a platform for development of appropriate model formalisms is established.

## Background

Finding regulatory interactions between cell products is one of the most important objectives of Systems Biology and has stimulated considerable research efforts [1-5]. DNA Microarray technology enables us to measure mRNA concentrations in a cell for a large number of genes at the same time. These levels can be viewed as a snapshot of the expression levels of genes under certain conditions. With a large enough set of snapshots, it should be theoretically possible to uncover the underlying gene regulatory network (GRN) [6].

One approach is to mathematically model the GRN and to find parameters of the model from available data. Once built, these models can be used to predict the behaviour of the organism under certain conditions, related to different treatments or diseases. Also, once the basic mechanisms of life are revealed, it has been postulated that it should be theoretically possible to create synthetic organisms, [7]. A large number of mathematical models and inferential algorithms have been developed. Generally, the process of modelling GRNs consists of a few main steps: choosing an appropriate model, inferring parameters from data, validating the model and conducting simulations of the GRN, to predict its behaviour under different conditions.

In order to model a GRN, genes are viewed as variables that change their (expression) values in time. Depending on the type of variables used, methods can be classified as discrete or continuous, deterministic or stochastic, or as hybrid methods, (using more than one type of variable). Two different approaches are distinguished in the literature, [1]: coarse-grained and fine-grained models, where the former contain less detail on interactions between genes. Usually, coarse-grained models rely on discrete variables, while fine-grained models are based on continuous variables. A GRN can be very large and may contain complicated interactions, so that a fine-grained model, typically, will have an enormous number of parameters to deal with. Both inference and analysis of this kind of model are difficult tasks, thus global, (high-level), analysis of the network, has its attractions. This includes coarse-grained models, such as Boolean networks, Bayesian networks, Petri Nets, rule sets: [8-20]. Other authors [21-33] have chosen to focus on detailed models, i.e. systems of differential equations, artificial neural networks, thermodynamic models, Hybrid Petri nets, *inter alia*, analysing only subnetworks of the entire GRN. A useful approach is to combine levels of detail, in a top-down or bottom-up approach, i.e. to move from a coarse to a more detailed model or vice versa [34-36]. Further information can also be found in [37,38], providing a collection of reverse engineering attempts and new challenges for researchers.

This paper concentrates on quantitative modelling of gene regulatory networks (GRNs) using DNA microarray data, as this is more informative than qualitative analysis of biological data. Although more sophisticated high throughput technologies have been developed lately, (such as next-generation sequencing), that may give more accurate results, [39], microarrays are still widely used, not only as an established, well-understood technology, but also as one for which appropriate automatic analytical tools exist. Even as newer methods become more pervasive, microarrays will remain both faster and less expensive for smaller genomes, (codeable as a single array). Consequently, use of the less-sophisticated technology is likely to persist for exploratory data analysis, (e.g. in identification of interesting features for in-depth investigation), at least in the medium term. Furthermore, from the viewpoint of this study, algorithms developed for one high throughput method can be applied to different measurement techniques, as the model of biological behaviour is the focus, not the data type as such. Our aim is to analyse different algorithms, used for such model inference, and to provide a comparison framework indicative of the advantages and disadvantages of each approach. We have chosen to analyse evolutionary algorithms (EAs) as suitable search methods for inferring GRN model parameters, as these are known to cope well with a large solution space, [40]. In particular, EAs can achieve good solutions from searching a relatively small section of the entire space, and have been widely used in genetic data analysis, (for an overview, see [41]).

Previous (pair-wise) algorithm comparisons for the methods analysed here have been reported, [23,42]. However, to provide a valid comparison of existing EAs for continuous models, algorithms assessed need to be applied not only to the same datasets, but also under the same framework. This work aims to achieve this and provide a consistent evaluation of ideas reported in the literature. The models used are not evaluated here, but only the algorithms that build models from data.

## Methods

In order to analyse the performance of evolutionary algorithms for model parameter inference, we have implemented seven different approaches and compared them on several datasets. These methods use different continuous fine-grained models to represent the GRN, and rely on EAs to find the model that best fits the experimental data. Further information on the implemented techniques can be found in [Additional file 1]. The algorithms were developed using EvA2, a Java framework for EAs [43] and the implementation and data sets used are available online. More information on downloading and using the framework can be found in [Additional file 2], [Additional file 3] and [Additional file 4] contain the source code of the implementation, while the datasets used are available as [Additional file 5].

### S-Systems

Generally, GRN models, based on systems of differential equations, express the change in the expression level of each gene in time as a function of the expression levels of the other genes, [1]. S-Systems are a special type of differential equation systems based on the power-law formalism and are capable of capturing complex dynamics, [44]. The disadvantages, compared to linear differential equations, (where regression techniques are easy to apply), are an increase in the number of parameters and a reduction in the available choices of reverse engineering techniques. The equations in S-Systems are of the form:(1)

In the case of GRN modelling, the two terms in Equation 1 correspond, respectively, to synthesis and degradation, influenced by the other genes in the network; *α*_*i *_and *β*_*i*_, the *rate constants*, represent the basal synthesis and degradation rate, and *g*_*ij *_and *h*_*ij*_, which indicate the strength of the influence of gene *j *on the synthesis and degradation of the product of gene *i*, are the *kinetic orders*. In real GRNs, it is, of course, possible that the expression level of a gene does not depend on the other genes, but only on its own concentration or that of metabolites or other external factors. Self regulation is modelled by S-Systems (parameters *g*_*ii *_and *h*_*ii*_), and metabolite concentrations can also be introduced in the model, when measurements are available.

Due to the fact that they are considered one of the most complete models for GRNs, S-Systems have received a lot of attention in the literature (e.g. [21,26,31,36,45]). This is also reflected in the work presented here, where six of the methods analysed use this type of model.

### Artificial Neural Networks (ANNs)

These are naturally-inspired models, which mimic the activity of the animal nervous system [46]. The network consists of units, called *neurons*, connected through weighted edges. By changing network topology and by using supervised learning algorithms to adjust the edges connecting neurons, an ANN is capable of approximating, theoretically, any possible function. Consequently, they are very well-suited for modelling purposes, especially when the underlying form of the model is unknown, which is also the case for GRNs. The disadvantage of this method is its complex topology; the regulatory causal interactions can not be extracted from the model, which can be a loss from the biological point of view (i.e. the 'black box' syndrome).

Two different ways of modelling GRNs with ANNs are common. The first one computes as the output of the ANN the *change in expression value*, with time, of one gene, while the other calculates the *expression value *itself at a certain moment in time. Inputs are the expression values of the regulators at the previous time point. The latter has been used here, (for one of the methods implemented), [35].

### Evolutionary algorithms

**Figure 1:**
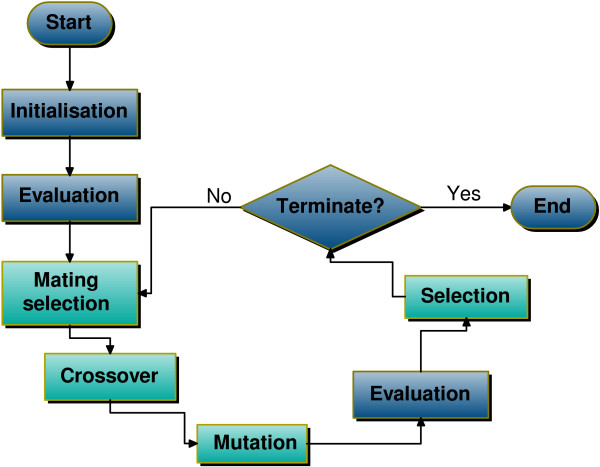
**Schematic view of evolutionary algorithms**. Some elements (light background) can be missing from different EA types.

EAs are a family of population based optimisation algorithms inspired by Darwinian evolution, sharing a set of common features. Included are: genetic algorithm, (GA), evolution strategy, (ES), genetic programming, (GP), evolutionary programming, (EP), differential evolution, (DE). Each maintains a population of solutions to the optimisation problem, (also called *individuals *or *chromosomes*), which evolve over a number of generations. The goodness of each individual, i.e. its *fitness*, is given by a function defined for the specific optimisation problem. Evolution is performed using genetic operators that depend on the specific problem and encoding, e.g (i) mutation, which modifies one solution from the population, to obtain a new one and (ii) crossover, which uses several parents to create a number of offspring. For each generation, a new set of solutions is produced from the previous population, either by replacing some parent individuals by children, or by performing fitness -based selection on all parents and children, (see Figure 1 for a schematic view).

Although these are common features of EAs, (representation, genetic operators, selection procedures, etc.), they are also the elements that differentiate one type of EA from the others. For instance, individuals of GAs are typically encoded as binary arrays, DE and ES use arrays of real numbers as an encoding for the solution, while GP evolve tree-encoded expressions. At the same time, these methods use different genetic operators, (applied to the different encodings), or use one *main *operator; (for instance, an ES does not perform crossover but only mutation on its individuals). Even given strict differences between each individual in the EA family of methods, the distinction has become fuzzier with time, as new hybrid approaches have appeared, such as the *real-encoded GA *used in this paper.

The generic methodology of fitting a GRN model to data using EAs involves a given model, a set of data, and evolution of the model parameters. For a population of parameters, representing different models, genetic operators are applied and the fittest individuals in the population are selected for the next generation. Usually, in the case of GRNs, the fitness function is defined as the difference between the observed data and the output of the model, (squared, or averaged over the data points). Since every model has its individual features, algorithm steps differ from one approach to another, but the main skeleton is usually preserved.

Here, we have implemented and analysed seven such algorithms: CLGA ([24]), MOGA ([47]), GA+ES ([34]), GA+ANN ([35]), PEACE1 ([23]), GLSDC ([48]) and DE+AIC ([26,31]) (more information related to these can be found in [Additional file 1]). Their analysis consists of two stages: (i) five hybrid EAs (GA+ES, GA+ANN, PEACE1, GLSDC and DE+AIC) were assessed for scalability, robustness to noise and performance with real microarray data, and (ii) two classical EAs (CLGA and MOGA), (the latter using multi-objective optimisation), were compared in a small-scale setting to evaluate the improvement introduced by the multi-objective approach.

Comparison of different EAs can be performed using several criteria. The most common are fitness value of best individuals at the end of optimisation and the number of fitness evaluations required for obtaining an observed fitness value. Robustness of fitness values and solutions obtained over multiple runs can also be analysed. Additionally, in this paper, a problem dependent criterion was used: the obtained solutions are also compared to the initial models (in the case of synthetic data), or to previous biological knowledge (for real microarray data). Robustness to noise analysis is performed by maintaining as fixed the number of fitness evaluations and other EA meta-parameters (e.g. mutation and crossover operators), and observing the decrease in fitness and solution quality. Scalability analysis involves increasing the number of fitness evaluations allowed and observing the quality of results obtained. The number of fitness evaluations was empirically chosen to allow the population to converge towards a stable fitness value (i.e. until only small improvements in fitness are observed). Table 1 lists the criteria used for comparison of implemented algorithms.

**Table 1:** This table defines criteria used for method evaluation

Criteria	Description
Goodness of data fit	Best/average Mean Squared Error (MSE) between data and model over a number of runs. This measures the ability of the model to reproduce the experimental data

Identified interactions	Ability of algorithm to qualitatively identify interactions (Sensitivity/Specificity). An interaction is taken to be identified if the corresponding parameter has an absolute value larger than zero. , Average values over multiple runs are used for comparison purposes.

Parameter quality	Best/average MSE between real parameters and algorithm solution over multiple runs. This measures the ability of the algorithm to find the exact parameters of a known model (important especially for underspecified systems.)

Robustness over multiple runs	Average variance of kinetic orders/rate constants over multiple runs

Robustness to noise	Performance of algorithm with noisy datasets: goodness of fit, identified interactions, parameter quality

Performance for real microarray data	Sensitivity/Specificity and goodness of fit when applied to real microarray experiments rather than to synthetic data

Scalability	Performance of algorithms with larger datasets, maximum dimensionality achieved, increase in running time and decrease in goodness of fit and identified parameter quality, (when moving from a smaller to a larger dataset)

Average running time	Over a number of runs.

Function calls	Average number of function calls required for the results obtained.

## Results and Discussion

In order to be able to evaluate our implementation on the chosen criteria, (Table 1), six datasets generated by S-System models of regulation and five for the artificial neural network (ANN) model were used. The models for two and five-gene S-System synthetic regulatory networks were taken from the literature, [24], and the ones for larger systems, (10, 20, 30, 50 genes), and for ANNs (5, 10, 20, 30, 50 genes) were randomly generated so that they conform to well known characteristics of real GRNs, i.e. scale-free sparse networks. Real GRNs are also known to display other characteristics such as modularity and feedback mechanisms, [49]. However, only sparsity is taken into account by the implemented methods, so using random sparse networks is a good indication of comparative algorithm performance. Nevertheless, we acknowledge that this could represent a limitation with respect to the significance of the synthetic experiments for the algorithm ability to reverse engineer the correct network from real data.

Robustness to noise was tested on the synthetic data for the five-gene networks to which 1%, 2%, 5% and 10% Gaussian noise was added to all values. The assumption of Gaussian noise has been used before in relation to gene expression data, [31,50], and, although it may not be true in all situations, it provides a good indication of the behaviour of the algorithm with real noisy data.

Ideally, in order to be able to build an S-System model, or to train an ANN, for a large scale network, a large number of measurements (time points) is required. This number increases further when data are noisy, [46]. However, in reality, due to the high cost of these experiments, only limited data are available. This leads to under-specification of the system, (i.e. the limited number of data points combined with the large number of parameters), which implies other parameter sets are able to reproduce the data (alternative models). Under these circumstances, EAs become a good alternative to other fitting methods, as they provide an efficient way of spanning the promising areas of the solution space. In order to simulate experiments with real data, we reduced the number of (synthetic) experimental time points used for inference to 60 for the 5-, 10- and 20-gene datasets, 80 for the 30-gene dataset and 125 for the 50-gene dataset. Through this, we aimed to obtain a balance between the need for an increased number of experiments and the cost of these experiments in the real setting.

As evolutionary algorithms are stochastic in nature, multiple runs were performed for each experiment. Multi-objective analysis was performed over 20 runs for each algorithm. The methods analysing the entire system were applied seven times on each dataset, while those using the divide and conquer approach were run five times for each of the first five genes, resulting in 25 runs per dataset. The quantitative results for the algorithms are displayed using notched box plots, [51], which show, for each result set, (obtained from multiple runs), the minimum, maximum, and quartile values. The notches around the median allow for a significance analysis of the differences between algorithms: if the intervals defined by notches around the medians do not overlap, then the observed difference between the medians is statistically significant; (we have used a 95% confidence interval in this paper). The graphs have been created using the Free Statistics Software from Wessa.net, [52]. The notches were reduced to the quartile limits, (whenever they exceeded these), in all the graphs displayed in this paper.

### Performance on small scale networks

**Figure 2:**
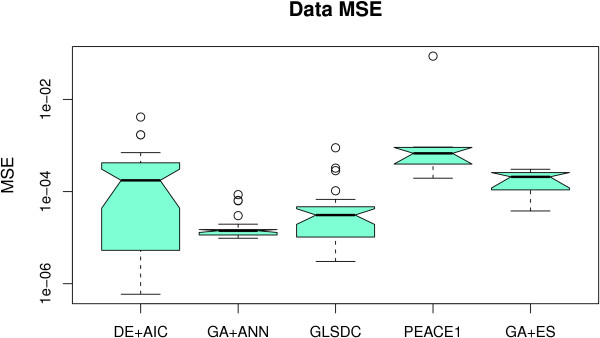
**Small-scale dataset - data fit**. Box plot displaying data MSE values for each algorithm with the 5-gene dataset. GA+ANN exhibits significantly better data fit, while PEACE1 has the lowest performance.

**Figure 3:**
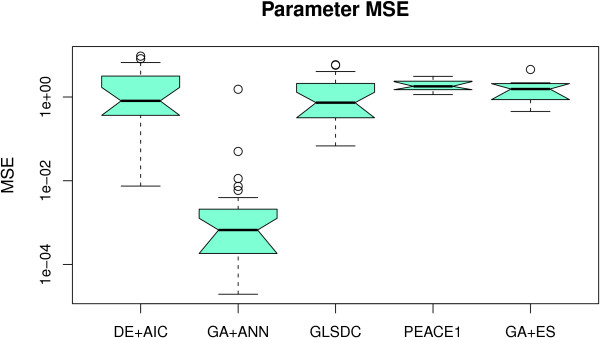
**Small-scale dataset - parameter quality**. Box plot displaying parameter MSE values for each algorithm with the 5-gene dataset. GA+ANN finds better parameters, conforming with data MSE values.

For a first analysis, we applied five algorithms to the five-gene synthetic dataset from [24]. We chose this benchmark dataset due to the fact that it has been already used to validate most of the methods we are comparing. At the same time, the small dimensionality allows for easier analysis of the EA parameters and for multiple runs to be performed. Figure 2 displays the box plots representing the data fit obtained by each algorithm, while Figure 3 presents the quality of parameters obtained over all runs performed. Table 2 contains numerical values for three more evaluation criteria (robustness of parameters obtained, sensitivity and specificity and fitness calls). Note that PEACE1 and GA+ES analyse all genes simultaneously, while the others find interactions one gene at-a-time. However, the numerical values for all the genes in the latter type of methods are used, allowing for a direct comparison between them.

**Table 2:** Performance of algorithms over multiple runs using the 5-gene synthetic dataset, under three criteria: robustness over multiple runs, qualitative interactions and number of function calls performed

Criteria	PEACE1	GA+ ES	GA+ ANN	GLSDC	DE+ AIC
Robustness (Kinetic orders/Rate constants variance)	0.25175/4.22818	0.4861/3.0170	0.07236	0.08449/2.0419	0.21534/6.41834

Identified interactions (Sensitivity/Specificity)	0.55384/0.82702	0.6483/0.8902	0.74074/0.8125	0.72307/0.67837	0.58461/0.81081

Function calls	1650000	3750000	2500 × 20000 ANN epochs	100000	275000

As Figure 2 indicates, all five methods demonstrate good performance in fitting the data (based on data MSE value). GA+ANN displays better fitness, followed by GLSDC, while PEACE1 performs least. The fact that the notches around the mean do not overlap proves these differences to be statistically significant at a 5% level. However, these are insufficient alone to choose a specific algorithm, as other options may exist and alternative model parameters may give a good fit to the data. Consequently, we provided (Figure 3) the parameter MSE values that show how close the resulting models are to the real one, which, in this case, are known, (i.e. how much does each parameter deviate, on average, from the real model). These values indicate again that the approach using the ANN model compares favourably to the rest. By analysing the values in Table 2, GA+ANN also appears more robust and better able to identify correct interactions. However, it should be noted that this model has fewer parameters compared to the others, (25 as opposed to 60), hence reducing the solution space for the EA, and, possibly, increasing algorithm performance.

Although methods using the S-System model display similar average performance, (according to the parameter quality criterion), GA+ES and DE+AIC obtain the best parameters overall (indicated by minimum values), while, (in sensitivity terms), GLSDC has higher value, indicating that the latter is more suitable for a quantitative analysis than the two former, which, despite finding parameter values close to the real ones, can miss smaller values.

Table 2 also supplies the number of function calls needed by the algorithms to achieve the performances above. These indicate the ANN approach to be faster, as, although each function call represents the training of an ANN, this is not very costly as these are small, due to the connectivity limit. PEACE1 requires a long running time, because of the numerous iterations needed to find all null parameters, and, given the low specificity, seems to miss the low ones. GA+ES also requires a large number of function calls, due to the overhead of running a new instance of an ES for each structure evaluation.

### Performance on noisy data

**Figure 4:**
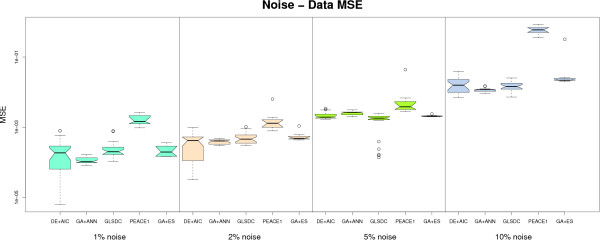
**Small-scale noisy datasets - data fit**. Performance of the 5 algorithms with noisy datasets in terms of data fit (data MSE). Algorithms displayed are, from left to right: DE+AIC, GA+ANN, GLSDC, PEACE1, GA+ES. An increase of MSE values with noise can be observed. PEACE1 displays lowest performance, while the rest of the algorithms are comparable under this criterion.

**Figure 5:**
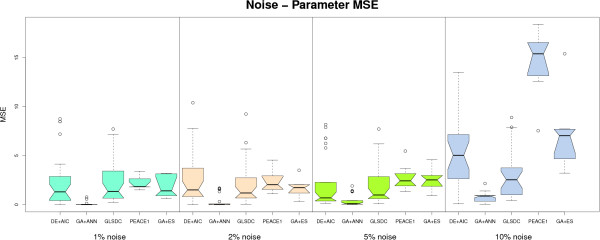
**Small-scale noisy datasets - parameter quality**. Performance of the 5 algorithms with noisy datasets in terms of parameter fit (parameter MSE). Algorithms displayed are, from left to right: GA+ANN, GA+ES, DE+AIC, GLSDC, PEACE1. GA+ANN exhibits (statistically significant) better parameters, while the rest of the algorithms display similar behaviour. At high level of noise (10%), GLSDC also performs better compared to the rest.

**Figure 6:**
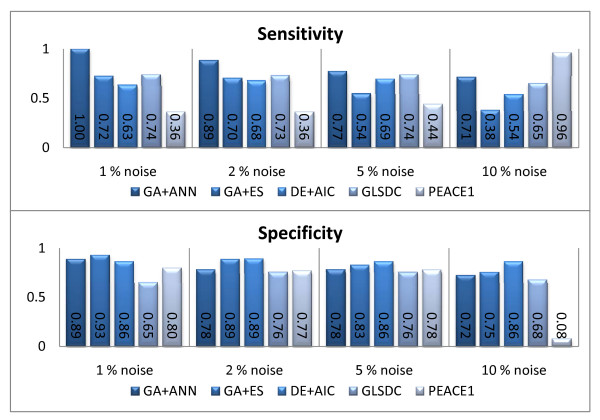
**Small-scale noisy datasets - identified parameters**. Performance, in terms of sensitivity and specificity, of the 5 algorithms with noisy datasets. Algorithms displayed are, from left to right: GA+ANN, GA+ES, DE+AIC, GLSDC, PEACE1.

An important feature for inferential GRN algorithms, in a real biological setting, is robustness to noise. We have analysed the behaviour of the algorithms implemented on noisy datasets, and the results are displayed in Figures 4 and 5, which show the evolution with noise of data fit and parameter quality, using the same type of box plots for significance analysis. Figure 6 shows average sensitivity and specificity values for the algorithms at the different noise levels.

The sensitivity and specificity criteria allow for a qualitative analysis of results. From the sensitivity point of view, the methods can be divided into three categories: with (1) stable sensitivity values (GLSDC, DE+AIC and GA+ANN), (2) decreasing sensitivity with noise (GA+ES), and (3) increasing sensitivity with noise (PEACE1). Specificity values, on the other hand, decline with noise for all methods, which is explainable by the fact that algorithms concentrate on finding null interactions, so the number of true negatives discovered decreases with noise. However, the first two categories seem to exhibit significantly better behaviour than the third. This explains why PEACE1 achieved a maximum sensitivity with maximum noise: a very small proportion of parameters were found to be null, so almost all genes were found to interact. This results in a large number of true positives, however, accompanied by a very large number of *false positives*, which is not desired here.

The quantitative perspective has been analysed using the two criteria in Figures 4 and 5. For PEACE1, both data and parameter fit are inferior to the rest, indicating limited ability to handle noise. However, only data MSE differences are statistically significant at all noise levels. The other four methods are stable and have comparable performance up to 5% noise, (favourable behaviour for real microarray data). Concerning the 10% noisy dataset, two trends can be indentified: GLSDC and GA+ANN decrease the data fit but preserve a good parameter quality (parameter MSE), while for DE+AIC and GA+ES both data fit and parameter quality decrease significantly. This means that the former set have the ability to find good parameters in spite of noise, while the latter over fit the noise in the data, implying low quality solutions. Good performance may be, in the case of GA+ANN, due to the nature of the ANN model, which has been proven to cope well with noise in multiple practical applications, [46], while GLSDC has a mechanism built in the local search phase that specifically handles noise.

In conclusion, the ANN model and the GLSDC mechanism for controlling noise seem to give good quantitative results even with a high noise rate. The best balance for sensitivity-specificity is achieved with GA+ANN, while GA+ES, DE+AIC and GLSDC exhibit the best qualitative behaviour with noise under the S-System model, (the former two find more null interactions, but miss some of the real ones and the latter finds most of the real ones but also adds some false positives).

### Scalability

**Figure 7:**
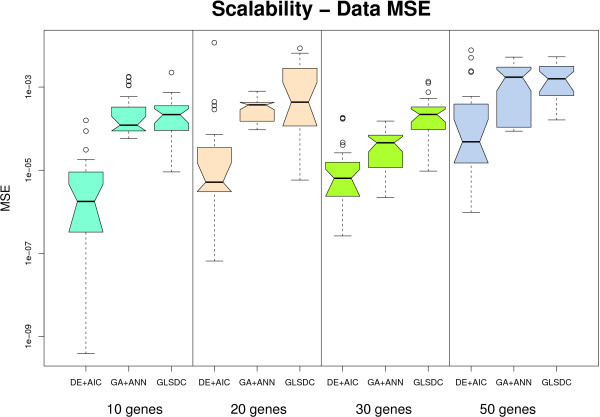
**Scalability - data fit**. Box plot representing data MSE with larger datasets. Due to poor performance with the 10- and 20-gene datasets, the values for PEACE1 and GA+ES are not displayed. DE+AIC exhibits (statistically significant at 5% level) better behaviour compared to the rest.

**Figure 8:**
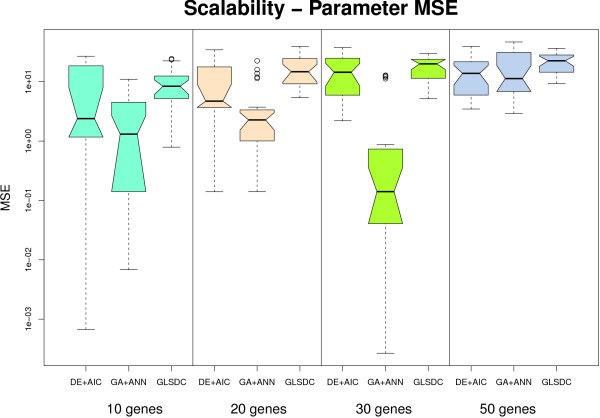
**Scalability - parameter quality**. Parameter MSE with larger datasets. Parameters identified by GA+ANN and DE+AIC are better than the rest up to 20 genes while for 30 genes only GA+ANN differs significantly.

**Figure 9:**
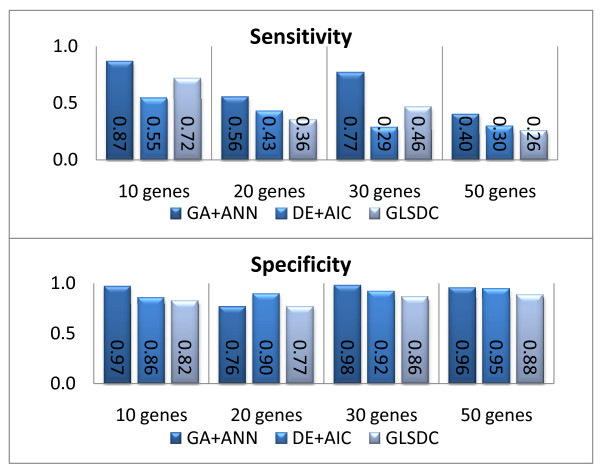
**Scalability - identified parameters**. Sensitivity and specificity for larger datasets (algorithms, from left to right: GA+ANN, DE+AIC, GLSDC).

Scalability analysis was performed on four synthetic datasets corresponding to four different networks: 10, 20, 30 and 50 genes. For these data, quantitative results using box plots are displayed in Figures 7 and 8, while the best qualitative results of all runs are shown in Figure 9. Given small sensitivity on the 10 and 20 gene datasets (approximately 0.1), and the dimensionality achieved by the authors themselves, (five genes), no further runs were performed with PEACE1 for the larger datasets. GA+ES was run on the 10-gene dataset with low performance (fitness 25 after 7,500,000 fitness calls, in 170 generations, during 47 hours), while on the 20-gene dataset, having doubled the allocated memory for the Java virtual machine, one generation lasted approximately 3 hours, and, after 35 generations (≈ 109 hours), the best fitness value was 1.4E11. This indicates that this method does not scale very well in a single CPU setting, and was thus discarded from the analysis. For the three methods that analyse one gene at-a-time, we performed experiments on a limited number of genes, (5), and averaged criteria values on them. The results obtained in this way are indicative of the performance of the methods for all the genes in the network. The rest of this section concentrates on these three methods.

Due to the characteristically low connectivity of the networks, all methods analysed displayed good specificity, (preserved for all system scales). However, the sensitivity values tend to decrease with the increase in size, which indicates that, for larger networks, these methods tend to set more and more parameters to zero, so that more interactions are missed. However, the number of false positives remains small. GA+ANN maintains a good qualitative performance up to 30 genes, while DE+AIC and GLSDC display good behaviour with the 10-genes dataset, but do less well as the size of the gene set increases. On the 50 gene dataset, all methods perform poorly, with respect to the sensitivity values.

In order to analyse the quantitative behaviour of the methods implemented, values for two criteria were provided: ability to reproduce data (Figure 7) and parameter quality (Figure 8). Considering the fact that each benchmark dataset has a different number of parameters to be inferred, of which most are zero, the parameter MSE displayed in Figure 8 is computed *per gene *rather than *per parameter*. Given the similar connectivity of the four different networks (3 to 5), this offers a good measure of parameter quality that neither depends on the number of genes in the network, which would have been the case if we had chosen the total squared error, nor is biased by the large number of null parameters usually discovered by the algorithms.

As Figure 7 indicates, all methods, except for those eliminated from this analysis after the first two experiments, (PEACE1 and GA+ES), display a good data fit for all datasets. However, DE+AIC exhibits a significantly better data fit at all scales.

GA+ANN achieves good parameter quality, (parameter MSE, Figure 8), up to 30 genes, confirming conclusions from the qualitative measures. DE+AIC exhibits a behaviour comparable to GA+ANN up to 20 genes, but displays lower parameter quality for 30 genes, possibly due to the limited data. The superiority of the first method could be partly due to the smaller number of model parameters, (half), compared to the other methods, the resulting system being less markedly under-specified than in the case of S-Systems and the solution space being reduced.

In conclusion, the method using the ANN model displays superior behaviour again with larger networks, while the methods that analyse the whole system at the same time failed to scale up for a single CPU situation. The other two methods behaved reasonably up to 30 genes, indentifying the most important interactions to enable them to closely reproduce the synthetic time series.

### Real DNA microarray data

**Figure 10:**
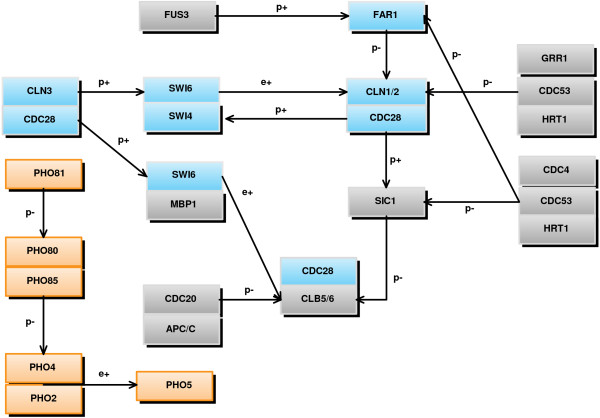
**Yeast cell cycle**. Pathway for Yeast cell cycle, retrieved from Kegg database, for the 24 genes analysed. The two coloured sets of genes correspond to the two small subnetworks, (6 and 7 genes), analysed separately. The connections between genes labelled with *e *represent known gene regulatory interactions, while the ones labelled with *p *represent known interactions between proteins that can activate or repress the activity of one or several proteins involved.

In order to assess performance of the chosen algorithms on real microarray data, the Spellman dataset [53] was used, which has become a benchmark for validating this type of method. This contains 18 time points measured during two *Saccharomyces Cerevisiae *cell cycles. The known interactions between genes and proteins were retrieved from the Kegg, [54], database for validation purposes. Three subsystems of this network were analysed; two small-scale (6 and 7 genes) and one medium-scale network, (24 genes), of which the former were subsets, (see Figure 10). The two small-scale networks contain the genes known to be involved, respectively, in the regulation of genes *CLN2 *and *PHO5*. The large-scale analysis focused on these two genes as well, to investigate how inclusion of additional genes, either not connected or distantly linked to the initial system, influences algorithm performance. The algorithms were applied five times for each gene under analysis.

**Figure 11:**
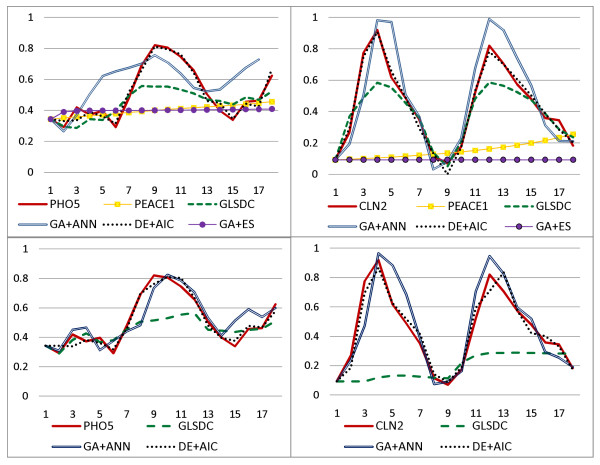
**Real data simulation**. Ability of algorithms to reproduce real data. The upper graphs display the real and the reproduced time series for the small-scale analysis, and the lower graphs for the medium-scale analysis.

**Figure 12:**
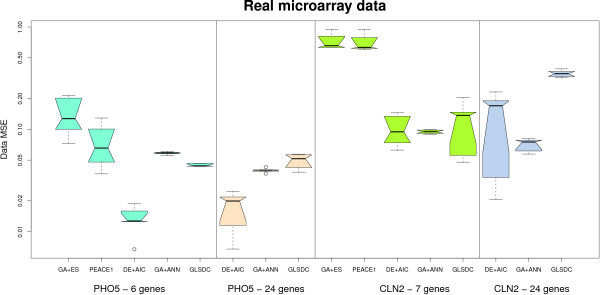
**Real data fit**. Box plot representing data MSE for experiments with real microarray data. For the first gene analysed (PHO5), DE+AIC displays best behaviour, while for the second (CLN2), both GA+ANN and DE+AIC perform comparably well. Due to scale limitations, experiments with PEACE1 and GA+ES were not performed for the 24-gene network.

Figure 11 displays the ability of each algorithm to reproduce the time series for the two analysed genes (best results obtained in multiple runs), while Figure 12 provides box plots for data MSE values. Even though, for the PHO5 experiment, the difference in MSE values, compared to GLSDC and GA+ANN, is not statistically significant, (as extracted from Figure 12), PEACE1 and GA+ES perform poorly in reproducing behaviour for the small networks (Figure 11). The small difference in data MSE values is due to the fact that the time series for GLSDC and GA+ANN are slightly shifted for this dataset, although overall behaviour is preserved. For the *CLN2 *experiment, both ability to reproduce time series and observed MSE values differ significantly. Given similar unsatisfactory performance on larger synthetic datasets, experiments with the 24-gene real dataset were not pursued with these two methods. Note that DE+AIC displays the best overall ability to reproduce the data, followed by GA+ANN and GLSDC. While GA+ANN and DE+AIC maintain good data fit for the larger dataset on both genes analysed, GLSDC fails to reproduce the data for *CLN2*, (Figure 11), and the MSE values increase significantly, (Figure 12).

Due to noise and the limited number of time points available, it is possible that, although a model is capable of reproducing the experimental data, the connections identified are false positives, and the model invalid. We have analysed the connections obtained, using data from the Kegg database and previous descriptions of the cell cycle from the literature, [55,56]. Table 3 displays the percentage of known interactions out of the total number of interactions identified by each algorithm in each experiment. The remaining percentage of the interactions predicted are clearly wrong, (either opposite sign or false connection). Both overall values and values corresponding to the fittest individual over multiple runs are presented, in order to facilitate a global view over algorithm performance. These known interactions considered correspond not only to transcriptional activation or repression, but also protein interactions, (e.g. phosphorylation, ubiquitination), that activate or repress transcription factors, hence influencing gene expression. For example, it is known that CLN3 and CDC28 work together to activate, (through phosphorylation), transcription factor SBF, (SWI4 and SWI6), which in turn activates gene *CLN1/2*; hence, *CLN3 *and *CDC28 *can also be considered as activators of these genes. The methods implemented often identify this type of interaction. Table 4 presents the average number of previously known direct interactions missed by each algorithm in each experiment.

**Table 3:** Percent of interactions identified by each algorithm that are known to exist previously, displaying average (overall) and best values over multiple runs

Experiment	GA+ANN	DE+AIC	GLSDC	GA+ES	PEACE1
6-gene PHO5	overall:92	overall:80	overall:41	overall:59	overall:25
	best:100	best:100	best:50	best:33	best:0

24-gene PHO5	overall:11	overall:15	overall:39	-	-
	best:0	best:14	best:40		

7-gene CLN2	overall:38	overall:40	overall:53	overall:36	overall:69
	best:50	best:40	best:60	best:40	best:75

24-gene CLN2	overall:29	overall:31	overall:18	-	-
	best:60	best:28	best:26		

**Table 4:** Average number of ovelooked important imediate interactions (from *SWI4/6 *for *CLN2 *and from *PHO4/2 *for *PHO5*)

Experiment	GA+ANN	DE+AIC	GLSDC	GA+ES	PEACE1
6-gene PHO5	1.67	0.4	0.4	1	1.5

24-gene PHO5	1.75	1.2	0	-	-

7-gene CLN2	0	0	0	0	1

24-gene CLN2	0.6	0.6	1.8	-	-

Note that, for some methods, the fittest individual identifies fewer interactions than the overall value, which confirms that good ability to reproduce data does not necessarily correspond to a model containing biologically relevant connections. Qualitative analysis indicates that, for the small networks, where all the genes are known to interact, the connections identified by the best-fitting methods are mostly correct. For the 7-gene experiment, two of the known interactions, (repression from *FAR1 *and activation from *SWI6*), have been consistently assigned parameters with the wrong sign, by *all the methods*, in multiple runs. This indicates noise interference, which explains lower values compared to the similar 6-gene experiment. GLSDC, however, seems to identify a number of interactions comparable to the 6-gene experiment, which confirms that it is more robust to noise than the others. GA+ES and PEACE1 also seem to correctly identify many interactions, but, the fact that the simulated gene values are highly dependent on the rest of the network, means they are unable to reproduce the experimental data.

Introducing more genes into the analysis triggers a different response from each method and gene analysed. In the *PHO5 *experiment, the percentage of correct interactions identified by GA+ANN and DE+AIC decreases markedly when analysing more genes, while the amount of overlooked direct interactions increases, although data fit remains very good or even increases, (from Figure 12, GA+ANN is significantly better in the second experiment compared to the first). This relies on connecting nodes that are not immediately linked in the real network, and, given that a large part of the added nodes may not be connected at all in reality, this leads to a low percentage of true positives. GA+ANN suggests a positive auto-regulation of *PHO5*, both with the small and large dataset, which can compensate for other missed interactions, and explain the improvement in data fit for the larger network. On the other hand, GLDSC maintains both quality of data fit, (though poorer than for the other two algorithms), and percentage of interactions, and adds fewer false interactions outside the *PHO *gene family, (connections from *SIC1 *and *APC/C*). This suggests that, when the added nodes are not connected to the existing ones, the algorithm is better at finding correct qualitative interactions, although fit obviously suffers.

In the second experiment, where most of the new nodes are connected to the initial network, GA+ANN and DE+AIC perform better both from the data fit and validity of interactions point of view. However, the number of false positives increases when moving to the larger dataset. GLDSC finds many effects of *PHO *genes on *CLN2*, but these are not biologically plausible. At the same time, when moving to the larger dataset, it correctly adds a positive effect from *FUS3*, that affects the gene through *FAR1*, but fails to identify the *SBF *complex (*SWI4/6*) as an activator. The fact that it does not succeed in identifying the main activation link explains the poor performance when reproducing the data. DE+AIC and GA+ANN preserve the connections from *SWI4*, *SWI6 *and *CLN3 *from one analysis to the other, but at the same time add some false connections to *PHO80*, *PHO4 *and *APC/C*.

All in all, the results indicate GA+ANN and DE+AIC as better choices when a continuous simulation of the system is required, with less concern for qualitative analysis of connections, (i.e. a black box approach). GLDSC seemed to identify correct interactions in most experiments, but, however, is not able to reproduce the data as well as the other two methods. The methods aiming to analyse all genes simultaneously displayed very poor performance in reproducing the data, although succeeded in qualitatively identifying some correct interactions for the small-scale datasets.

### Single versus multi-objective optimisation

As CLGA ([24]) and MOGA ([47]), described in [Additional file 1], were found not to be suitable for large networks, they were compared only to each other in a small network setting, i.e. a two-gene GRN. The approach used in MOGA is to split the squared error fitness of CLGA into separate objectives for each gene. Hence, in our experiments, we had 2 objective functions to minimise. The aim of this experiment is to compare CLGA with this multi-objective (MO) approach and to identify the benefits of introducing fuzzy domination. The results of this experiment should be indicative of the improvement of other, more advanced EA approaches, when using MO optimisation.

**Figure 13:**
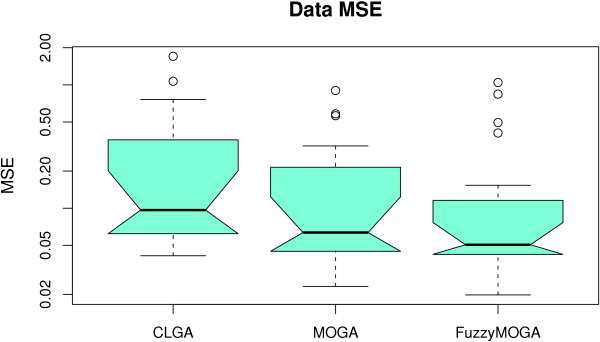
**Multi objective optimisation - final data fit**. Final data MSE for CLGA, MOGA and Fuzzy MOGA on the 2-gene dataset. The difference observed is not statistically significant at a 5% level.

**Figure 14:**
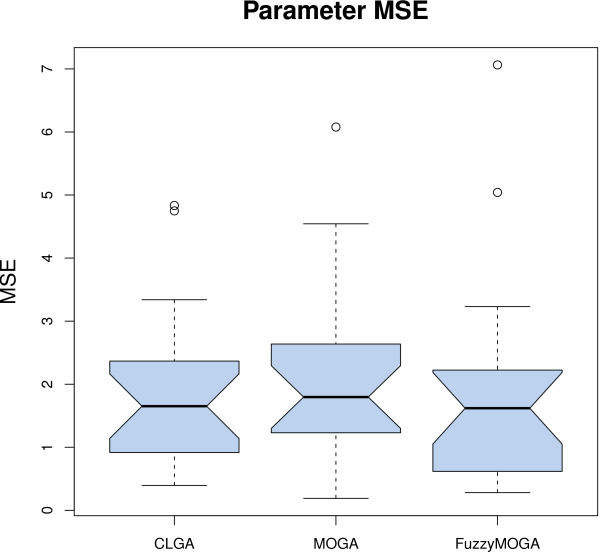
**Multi objective optimisation - parameter quality**. Parameter MSE for CLGA, MOGA and Fuzzy MOGA on the 2-gene dataset.

In order to ensure the validity of our comparison we performed twenty 100,000-fitness call runs for each of the three algorithms and the results are summarised in Table 5 and Figures 13 and 14. The averaged values in the table have been computed after eliminating the worst two and best two of the results for each algorithm.

**Table 5:** Performance of classical vs multi objective real-coded GA over 20 runs using the 2-gene synthetic dataset

Criteria	CLGA	MOGA	Fuzzy MOGA
Goodness of data fit (Best/Average SE)	0.04115/0.209183826	0.023202753/0.14008799	0.019817555/0.10705668

Parameter quality (Best/Average SE)	2.368969422/10.25508788	1.138802292/11.22558038	1.685895101/9.676239684

Robustness (Kinetic orders/Rate constants variance)	0.324876643/1.107014477	0.320798581/4.085473222	0.279334243/1.518127766

Average running time	187.6s	302.8s	300.6s

**Figure 15:**
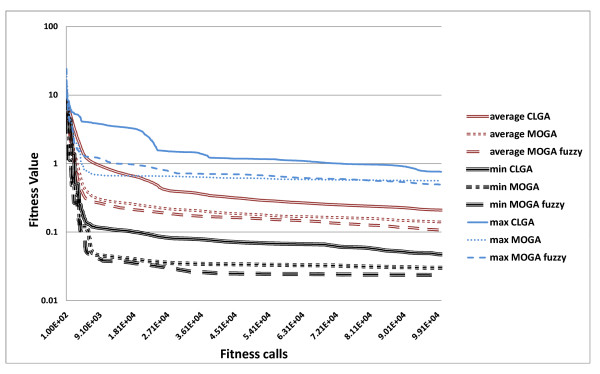
**Multi objective optimisation - fitness evolution**. Comparison of CLGA, MOGA and Fuzzy MOGA on the 2-gene dataset during all generations.

Figure 15, which shows the average, minimum and maximum squared error between the data and the model for the 20 best individuals in each generation, (one for each run), indicates that the MO algorithms perform better in terms of goodness of fit (the models found simulate the time series better than the CLGA). However, Figure 13 indicates this difference is not statistically different at a 95% confidence level. A t-test shows that the improvement is significant only with 85% confidence. Similarly, although minimum values found for parameter MSE are better for the multi-objective approaches, the differences are not statistically significant. Note, however, (Figure 15), that the two multi-objective approaches converge faster. This observed difference is confirmed by a t-test performed on fitness values obtained after 20 000 iterations (a fifth of total optimisation), that resulted in *p *0.02 when comparing the single with the multi-objective approaches. However, no significant improvement is introduced by fuzzy dominance selection in this case.

A more general observation is that, if we perform two rankings of the 20 solutions obtained, (by goodness of fit and parameter quality, respectively), results differ, for all three methods. So, improved fitness does not necessarily mean better parameters. This indicates that some parameters may be more important than others, so that a slight change in the values of the more meaningful ones strongly influences the ability of the model to reproduce the data. Another argument for this is the observed difference between the robustness of kinetic orders and that of rate constants, which suggests that the latter can vary more without affecting goodness of fit too much. These observations also suggest that alternative models are possible, so that more precise discrimination is needed.

In conclusion, we have shown that, splitting the squared error objective into smaller sub-objectives, for a MO approach, significantly speeds up convergence for EAs. Nevertheless, after a large number of iterations, final results are comparable. This could be due to the fact that this approach forces the algorithm to fit all parts of the time series at the same time, instead of allowing it to converge more slowly by improving only some of the objectives, which is an advantage, especially when dealing with large dimension problems as performing a very large number of iterations is not viable. This suggests that, even when analysing only one gene at-a-time, we can still split the time series into shorter parts, to speed up convergence in a MO setting. Further analysis, to investigate to what extent this objective division is useful and at what point the overhead becomes greater than the gain, would be valuable.

### Divide et impera?

Two different approaches for GRN model parameter inference are advocated in the literature: finding relations for the entire network, [24,34,57], or analysing a single gene at-a-time, [26,31,35,48]. Among the methods implemented in this work, three use the latter (GLSDC, GA+ANN, DE+AIC). An obvious question is which of the two approaches is more reliable.

The argument *in favour of division *found in the literature is *increased scalability *due to decrease in number of parameters, (linear instead of quadratic dependency on the number of genes in the network), and ease of solution evaluation, as only the time series for the current gene needs to be simulated. However, these arguments do not take into account the fact that this method has to be iterated for all genes, so, ultimately, the number of parameters and the number of simulated time series is the same, (no significant increase in running time or computational power needed). Also, when simulating one series at-a-time, the values of the rest of the genes are considered to be those of the experimental data. However, the effect of the current gene on the others is not taken into account, and this can give the impression of finding a good solution when, in reality, the difference between the data and the simulation in a whole system setting could be larger. This effect is exacerbated for real noisy data. In order to compensate for this disadvantage, a complete network analysis can be performed, to fine tune the parameters obtained for each gene in each sub-problem.

In order to avoid the resource problem and be able to scale up even when analysing the entire network simultaneously, parallelisation is clearly desirable. In a parallel setting, division loses its advantages, becoming less viable than the complete network analysis, which can be parallelised in a more convenient way, to avoid simulating only part of the network when evaluating individuals.

During our experiments, division proved to be more useful when analysing real data, statistically significant differences being observed in one of the small scale experiments. Nevertheless, in both of these experiments, probably due to noise, the two methods analysing the complete networks failed to reproduce the time series, even for a small number of genes. However, a more detailed analysis, in a multi-CPU setting, is required with respect to their behaviour with real microarray data.

### Inclusion of prior knowledge

Although microarray data provides measurements for a large number of genes, the number of time points available is usually not enough for a quantitative analysis of the underlying GRN [1]. A very large pool of biological knowledge and prior information on possible interactions exists in the literature, but the effort made to integrate these has been sparse so far. EAs in general, and in particular these approaches implemented, have the benefit of flexibility in terms of adding prior information to the optimisation process. This can be done at several stages, such as initialisation, fitness evaluation, mutation or crossover, etc. An example of integrating biological knowledge in the algorithms implemented is using the sparsity of the GRN, (as part of the fitness function [23,26,48], by local search [26] or through nested optimisation [34,35]). Further improvement could be introduced in these algorithms by adding additional knowledge, (a future research direction that we plan to pursue).

For instance, previously known interactions could be introduced during initialisation, and links maintained until the end of the optimisation (similar to [45]). In the same manner, statistical information on possible interactions, obtained by preliminary (pair-wise) analysis of gene expression data, [58], can be integrated in the optimisation to accelerate convergence and improve solutions. In particular, the nested optimisation algorithms implemented, (GA+ES, GA+ANN), could benefit from this type of knowledge, as structure is already separated from parameter values during optimisation, and this could help avoid evaluation of completely impossible structures, (implying a new ES instance or Backpropagation, which are time consuming).

Similarly, binding affinities and gene sequence structure could boost performance for the algorithms. This type of knowledge has been used before with a Bayesian model [59], however, not with EAs, to our knowledge. The prior information can be used both in initialisation and during fitness evaluation (edges connecting genes with binding affinities could add to the fitness of the individual). This can be easily introduced in any of the methods presented here.

Producing long time-series experiments is very costly and not feasible for most laboratories. However, short series from different sources, but describing the same process, are available. Nevertheless, no efforts have been made to combine these for model inference. It is possible, by using adequate normalisation techniques, to combine these heterogeneous datasets, and be able to model the common features. The same gain could be obtained by fitting different replicates of the same experiment as a separate time series. This should also increase the ability of algorithms to handle noise, as, by combining data with heterogeneous perturbations, over fitting of the noise is reduced.

## Conclusions

This article presented a comparison of existing methods of inferring parameters for continuous models of gene regulation, based on DNA microarray data. We have implemented seven algorithms (CLGA [24], MOGA [47], GA+ES [34], GA+ANN [35], PEACE1 [23], GLSDC [48] and DE+AIC [26,31]) and compared these for different time series data sets in order to analyse their behaviour under a common framework. The main aim was to identify which methods perform better under different GRN criteria, in order to assess directions for improvement.

A first observation derived from our experiment is that pure evolutionary algorithms are powerful enough to analyse only very small-scale systems, as found for CLGA and MOGA. In order to increase power, hybridisation is typical and results show that hybrids are suitable for larger networks. We have shown that the methods implemented can achieve good performance up to 30 genes.

We applied five of the methods to real microarray experimental data, which had been considered only for DE+AIC and GA+ANN, to date, and, for the latter, in a discrete setting only. GA+ANN and DE+AIC proved to be capable of closely reproducing the original time series even for a larger dataset, (statistically significant differences were observed), while identifying, at the same time, some of the known interactions in the data. GLSDC also identified known interactions, but had limited ability to reproduce the data. The two methods analysing the entire network simultaneously, (GA+ES and PEACE1), failed to reproduce real data, which suggests that existing methods are not as yet capable of simulating the entire network in a real experimental setting, even when analysing small-scale systems.

We have shown that splitting the evolutionary algorithm objective into smaller sub-objectives, (for a multi-objective approach), speeds up convergence. This suggests that, even when analysing only one gene at-a-time, we can still split the time series into shorter parts. Furthermore, we believe that using multi-objective optimisation along with a hybrid approach can improve learning performance.

Importantly, it should be noted that parallel implementation of the evolutionary algorithms is necessary, (supported by literature, [31,60], as well as by our experiments). Hybrid methods are computationally expensive and, although these work well with small networks on a single machine, they tend to become less efficient for larger networks, especially those analysing the entire network simultaneously. In order to achieve scalability, parallelisation can be performed at several levels, ranging from individual evaluation to iterations and division of the entire problem into sub-problems.

A very important issue with gene regulatory network inference from microarray data is both the limited and noisy nature of these data. This indicates the need to use time-series from different sources and other types of biological data, (widely available), in order to underpin relationships between genes. These data include (1)ChIP Chip data and binding affinities, which identifies which proteins bind to which genes, indicating possible interactions, (2) knockout microarray experiments, which allow for mutant behaviour to be analysed, (3) protein-protein interactions, which indicate groups of co-regulated genes, (4) miRNA interference data, which indicates other causes for a gene to be under-expressed. These data can be potentially included in the evolutionary algorithm in a multi-objective setting, in order to speed up convergence.

## List of abbreviations

EA: evolutionary algorithm; ES: evolution strategy; GA: genetic algorithm; GRN: gene regulatory network; ANN: artificial neural network; MSE: mean squared error; MO: multi-objective; GA+ES: method nesting a genetic algorithm with an evolution strategy; AIC: Akaike's Theoretic criterion; DE+AIC: method using differential evolution as a search strategy, and AIC-based fitness; GLSDC: method using genetic local search; PEACE1: iterative algorithm based on GA; CLGA: classic GA; MOGA: multi-objective GA; GA+ANN: method using an ANN as a model and GA for parameter inference.

## Authors' contributions

AS implemented the methods in Java and applied them to the different datasets. All three authors participated in the design of the experiments, interpretation of results, composition and drafting of the manuscript. All authors read and approved the final manuscript.

## Supplementary Material

Additional file 1 **Implemented evolutionary algorithms for gene regulatory network inference**. This PDF file gives details on the 7 algorithms implemented and analysed here. Click here for file

Additional file 2 **Using the framework**. This PDF file provides information on downloading and using the Java implementation for algorithm comparison. Click here for file

Additional file 3 **EvA2 framework**. This archive contains the code and resources published by EvA2 authors (Minimum corresponding code). More details on how to use it can be found in Additional File 2. Click here for file

Additional file 4 **Algorithm implementation**. This archive contains the code for the seven methods implemented (Corresponding application code). More details on how to use it can be found in Additional File 2. Click here for file

Additional file 5 **Data sets**. An archive containing the datasets used for the experiments presented here. Click here for file
