# Supplementary material for: Comparison of evolutionary algorithms in gene regulatory network model inference
Source: BMC Bioinformatics. 2010 Jan 27;11:59. doi: 10.1186/1471-2105-11-59 (PMC2831005; doi:10.1186/1471-2105-11-59)
Supplement: Additional file 1 — Implemented evolutionary algorithms for gene regulatory network inference. This PDF file gives details on the 7 algorithms implemented and analysed here. [file 1471-2105-11-59-S1.PDF]

Additional file 1: Implemented evolutionary algorithms for gene regulatory network inference

Classic GA (CLGA)

Introduced by [1], this algorithm optimises parameters for an S-System model using a simple real coded GA. The $n(2n+2)$ parameters $(\alpha_i, \beta_i, g_{ij}, h_{ij})$ of the system are encoded in a $n \times (2n+2)$ matrix of real values, representing the individuals in the algorithm. Classical uniform crossover and mutation operators on vectors of real values are used. The algorithm minimises the difference between the microarray expression values and the expression values generated by the model.

$$fitness = \sum_{i=1}^{n} \sum_{t=1}^{T} \left(\frac{x_i(t) - y_i(t)}{x_i(t)} \right)^2 \tag{1}$$

where $x_i(t)$ is the expression value of gene i at time t, observed in real experiments, and $y_i(t)$ is the expression value of gene i at time t generated by the model. In order to evolve sparse networks[1], the parameters falling below a pre-determined threshold are automatically set to zero in each generation. This was the first attempt to use EAs for this problem, and opened the way for many other approaches. Although its performance, finding parameters for 2 genes in a GRN, has been overtaken, this algorithm is retained in order to compare it to a multi objective approach.

MOGA

Multi objective optimisation is known to be more effective than combining the set of functions into a single one, as it forces all individual fitness values to be close to the optimum. This approach was applied to GRN inference by [2], which, rather than adding errors (as in CLGA), used those corresponding to *each* gene expression series as a *different objective.* Furthermore, the concept of *fuzzy domination* was introduced, which gives a continuous aspect to individual domination in multi objective optimisation. The Simplex algorithm was used to create part of the offspring for each generation. We have implemented this approach, apart from the Simplex algorithm. This was omitted in order to test whether the multi-objective approach *itself* improves the search performance in this problem setting and to determine how fuzzy dominance-based selection affects its performance, without hybridising the search method as well. NSGAII tournament selection (implemented in EvA2) was used for MOGA and fuzzy dominance-based tournament selection for the Fuzzy MOGA. The MO algorithms use an archive of a maximum of 50 individuals belonging to the Pareto front that are fed to the next generation, to implement elitism.

GA+ES

One of the more advanced and recent approaches to applying EAs to GRN modelling divides the search into two phases: structure and parameter search. This is based on the sparsity of the networks: as most of the values in the connection matrix are null, there is no need to search for all the parameters, but only

[1] GRN are known to be sparsely connected, as each gene has a small number of regulators. This makes the matrix that encodes the solution sparse: many values are null, and this fact is used in this approach.

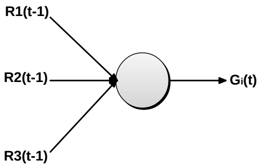

Figure 1: ANN Topology in GA+ANN: represents how expression value of a gene G at time t is computed from the expression values of its regulators, R_1, R_2 and R_3 at time $t-1$ through a single layered ANN.

for those found to be non-null. Consequently, one phase of the algorithm looks for the non-null parameters, and the other finds the real values for them. These two phases are nested into each other, the second one being similar to a local search performed on the individuals of the first. The first method of this kind we have implemented is the one introduced by [3]. Here, a GA finds the connection matrix with elements in $\{0, 1\}$ and then an ES is employed to find the S-System parameters for the connections represented by the individuals of the GA. The fitness of the GA individuals is given by the best individual in the ES. The method was reported to have achieved parameter determination for a 20-gene artificial GRN.

GA+ANN

The second method implemented, which involves nesting the structure and the parameter search phases, is [4]. This is a neural-genetic hybrid that replaces the S-System in GA+ES with an ANN. Here, the structure search uses a different representation: a binary encoded set of real values, rather than the characteristic vector[2] used in [3]. The ANN, which is a perceptron, takes as input a subset of genes and learns, through Backpropagation, the parameters for regulation, (in order to find regulation strengths from each gene to the gene under analysis), then outputs the error of the resulting model as fitness for the GA individual. Figure 1 shows the ANN topology used by this method. The fact that it is single layered allows for inference of causal relationship from the topology.

PEACE1

Initially, most methods that took into account the fact that gene networks are sparse, forced parameters that fell below a certain threshold to zero, (e.g. [1]). An additional way of generating sparse networks was introduced in [5], extending CLGA. The fitness function was modified so that the algorithm tries to minimise the squared error of the model (first term in Equation 2) , while penalising the models with high connectivity (second term):

$$fitness = \sum_{i=1}^{n} \sum_{t=1}^{T} \left(\frac{x_i(t) - y_i(t)}{x_i(t)} \right)^2 + cnT \sum_{j=1}^{n} (|g_{ij}| + |h_{ij}|) \quad (2)$$

Here, c is a constant that balances the two terms in the fitness function. Simplex crossover is used and a two-stage gradual optimisation strategy is employed: initially, the GA is run several times to find multiple models that fit the data. Using the resulting models, a new instance of the GA is initialised and evolved towards a model that combines parameters from local solutions into a better global one. During all generations, the parameters that fall below the threshold are set to zero, in order to simplify the simulation process when evaluating an individual. This improved model is analysed and the null parameters are fixed at zero for the next iterations. This double optimisation (feeding one GA with the results of other GAs), is needed in order to avoid setting necessary parameters to zero. Afterwards, a new set of GA instances are employed

[2] A characteristic vector encodes a subset of a finite universe, where, for each element in the universe, a boolean value is used that denotes whether that element is in the subset or not.

to find local solutions, taking into account the fixed parameters. This procedure is iterated until no more parameters need to be suppressed or a maximum iteration number is reached.

GLSDC

GLSDC, [6], evolves parameters for one gene at a time, using a skeletalising term similar to [5]:

$$fitness_i = -\sum_{t=1}^{T} \left(\frac{x_i(t) - y_i(t)}{x_i(t)} \right)^2 - c \sum_{j=1}^{n-I} (|G_{ij}| + |H_{ij}|) \tag{3}$$

Here, I is the maximum connectivity parameter and G_{ij} and H_{ij} are elements of two sets containing g_{ij} and h_{ij} in ascending order of their absolute values. The approach differs from that of [5] in that only the chromosomes that contain more than I activators and I repressors are penalised by the second term of the fitness function, and only the network connections with the lowest weights are included in the penalty term, (as those with high weights are taken to be the correct interactions).

One of the most costly parts of the EA implementations previously presented is the evaluation of individuals, as they require simulations of the model. GLSDC minimises this problem by splitting evolution into two stages: local search and convergence stage. During the convergence stage, crossover and mutation are applied to the population, similar to a classic GA, but no evaluation is performed, so that evolution is faster. During local search phase, a set of restrictions, (inequalities involving model parameters), derived from data, are tested on the individuals in the current population. In case the conditions are not met for one individual, a local search based on quadratic programming, [7], is performed in order to find the closest parameters, (Euclidean distance), that do satisfy the inequalities. For the rest of the individuals, i.e. those conforming to the restrictions, Powell's local search, [8], is performed, to find a solution with a better fitness.

DE+AIC

DE+AIC, [9, 10], introduced a further improvement with respect to the penalty expression that forces the algorithm to evolve sparse models. Instead of ordering g_{ij} and h_{ij} in sets G_{ij} and H_{ij}, as seen in GLSDC, these are combined in only one ordered set K_{ij}. The fitness function no longer measures the closeness of the data generated, by the model, to the experimental data, but uses an information theoretic criterion: Akaike's Information Criterion (AIC). This measures the likelihood of the data under a specific model and penalises those models which describe the data using a large number of parameters. The algorithm evolves parameters for one gene at a time. Considering all the modifications specified above, the fitness function, which the GA needs to minimise, becomes:

$$fitness_i = -2\Lambda + 2\phi_i + c \sum_{j=1}^{2n-I} |K_{ij}| \tag{4}$$

where Λ is the likelihood of the data under the model encoded by the current chromosome, ϕ_i is the number of parameters corresponding to gene i and I is the maximum connectivity.

The search heuristic in [9] is Trigonometric DE. For each generation, a local search is operated using Hill Climbing[3], [11], to further skeletalise the models, (obtain sparser realisations), encoded by the best individual and one selected randomly from the population. The procedure parses the set, K, of sorted kinetic orders,(g_{ij} and h_{ij}), from the smallest to the largest. At each step, it sets the current kinetic order to zero, and, if the newly identified individual is better that the one before the change, it replaces the old

[3]An optimisation heuristic that starts with one solution and iteratively modifies it to obtain a better one. The modification is usually done with operators similar to GA mutation. The old solution is replaced by the new one only if the latter is better. The search stops either after a certain number of mutations or when the solution does not improve any more, within a specified tolerance.

one. This is repeated until all the kinetic orders have been changed. In a sense, this local search is similar to Powell's method, [8], as it searches each dimension at a time. This approach may miss some improvements that may happen only when two or more parameters are null *at the same time*, but, however, setting one parameter at a time to zero brings no fitness increase. Given that it is used as a local search technique in a more ample search, this is not a major drawback.

References

1. Tominaga D, Okamoto M, Maki Y, Watanabe S, Eguchi Y: **Nonlinear Numerical Optimization Technique Based on a Genetic Algorithm for Inverse Problems: Towards the Inference of Genetic Networks**. In *GCB99 German Conference on Bioinformatics* 1999:101–111.

2. Koduru P, Das S, Welch S, Roe JL: **Fuzzy Dominance Based Multi-objective GA-Simplex Hybrid Algorithms Applied to Gene Network Models**. In *Genetic and Evolutionary Computation - GECCO 2004* 2004:356–367.

3. Spieth C, Streichert F, Zell NSA: **Optimizing Topology and Parameters of Gene Regulatory Network Models from Time-Series Experiments**. In *Genetic and Evolutionary Computation - GECCO 2004* 2004:461–470.

4. Keedwell E, Narayanan A: **Discovering gene networks with a neural-genetic hybrid**. *Computational Biology and Bioinformatics, IEEE/ACM Transactions on* 2005, **2**(3):231–242.

5. Kikuchi S, Tominaga D, Arita M, Takahashi K, Tomita M: **Dynamic modeling of genetic networks using genetic algorithm and S-system**. *Bioinformatics* 2003, **19**(5):643–650, [http://bioinformatics.oxfordjournals.org/cgi/content/abstract/19/5/643].

6. Kimura S, Hatakeyama M, Konagaya A: **Inference of S-system models of genetic networks using a genetic local search**. *Evolutionary Computation, 2003. CEC '03. The 2003 Congress on* 2003, **1**:631–638 Vol.1.

7. Boot JC: *Quadratic programming. Algorithms - Anomalies - Applications.* Rand McNally & Company 1964.

8. Press WH, Flannery BP, Teukolsky SA, Vetterling WT: *Numerical Recipes in C: The Art of Scientific Computing.* Cambridge University Press 1992.

9. Noman N, Iba H: **Inference of genetic networks using S-system: information criteria for model selection**. In *GECCO '06: Proceedings of the 8th annual conference on Genetic and evolutionary computation*, New York, NY, USA: ACM 2006:263–270.

10. Noman N, Iba H: **Inferring gene regulatory networks using differential evolution with local search heuristics**. *IEEE/ACM Transactions on Computational Biology and Bioinformatics* 2007, **4**(4):634–647.

11. Chambers LD: *The Practical Handbook of Genetic Algorithms: Applications, Second Edition*, Boca Raton, FL, USA: CRC Press, Inc. 2000 .
