# Supplementary material for: Comparison of evolutionary algorithms in gene regulatory network model inference
Source: BMC Bioinformatics. 2010 Jan 27;11:59. doi: 10.1186/1471-2105-11-59 (PMC2831005; doi:10.1186/1471-2105-11-59)
Supplement: Additional file 2 — Using the framework. This PDF file provides information on downloading and using the Java implementation for algorithm comparison. [file 1471-2105-11-59-S2.PDF]

Additional file 2: Downloading and using the code

This document is a tutorial explaining how to download and run the implementations of the seven methods compared. This code is distributed under GPL public licence. In order to be able to use it, Java 1.6, which can be downloaded from http://java.sun.com/javase/downloads/index.jsp, is required.

Downloading and installing

EvA2 framework, [1], was used for implementing the seven example algorithms from the literature. The latest version of this software can be downloaded from: http://www.ra.cs.uni-tuebingen.de/software/EvA2/download.html. However, due to modifications from the previous version, our code is not compatible to the new version. In consequence, we are making available both the previous version of EvA2 and the sources for the implemented techniques, as 'combined work' as stated in the LGPL licence document provided with the EvA2 framework. The framework source code used (Minimal Corresponding Source) is made available in Additional File 3, while the additional code implementing the methods, (Corresponding Application Code), is available in Additional File 4. To use this code, unpack the archives in the same folder and, using a Java IDE, create a new project with *src* as source folder.

The application uses JAMA, a Java Matrix Package that can be downloaded from http://math.nist.gov/javanumerics/jama/Jama-1.0.2.jar, for matrix operations, and Mosek, http://www.mosek.com/, for Quadratic Programming. A free academic licence for Mosek is available on the indicated website. Once the two dependencies are downloaded and installed, add *Jama-1.0.2.jar* and *mosek.jar* to the project classpath. The project is now ready to be run (main type is *eva2.client.EvaClient*). Please make sure a copy of the resources folder exists in *bin*.

Using the application

Once the IDE project is set up, it can be run and the implemented algorithms tested on different datasets. These are implemented by extending the class *AbstractOptimisationProblem*, as designed in EvA2. The algorithms are identified by the authors' names and year of publication, and the corresponding implementations can be found in the package *eva2.server.go.problems.dcu*. Package *ie.dcu.modsci.grn.utils* provides utility code for the algorithms implemented.

In order to run the algorithms, a file containing gene expression data is required. Additional file 5 contains an archive with the data used in the paper. Please unpack this in the project folder created.

The EvA2 user interface is used to select the problem and the optimisation technique desired. In order to make a new selection for one of the elements (e.g. optimiser, problem, terminator), click on the textbox containing the current selection and choose a new option from the dropdown. The interface allows you to set parameters for your new element. Once all the elements are correct, optimisation is started by clicking on the 'Start' button. The rest of this document describes parameters available for all methods implemented. For more information on how to use EvA2, please read the manual provided by the authors.

CLGA

- Problem. To test this method, [2], the Problem eva2.server.go.problems.dcu.Tominaga99GrnFromMad has to be selected using the GUI. Problem parameters:

- KineticOrdersRange. This parameter is common to all methods using the S-system model. It has to be an array of size two indicating the search interval for kinetic orders (g_{ij} and h_{ij}).
- MadFileName. The name of the file containing microarray data (gene expression data). This file should contain the number of genes and number of time series on the first line. For each of the time series, a line mentioning the number of time points has to be inserted, then a new line for the time spans between points, followed by the data itself (each line contains the expression values for all genes at the current time point). The format of the file can be seen in the examples provided in Additional File 5. This parameter is common to all problems described here.
- ModelType. This parameter allows choosing between the S-System and a linear model for some problems. 0 stands for S-System, 1 stands for linear model.
- RateConstantsRange. Similar to KineticOrdersRange, this parameter defines the search interval for rate constants (α and β). This parameter is common to all problems using the S-System model.
- SkeletalisingThreshold. When parameters in the model are lower than this value, they are set to 0. This parameter is common to all problems described here.
- TemplateIndividual. Allows changing attributes for the individuals in the population. Clones of the template individual are used for population initialisation. This parameter is common to all problems described here.

- Optimiser. The optimiser used for this problem is GA with elitism and tournament selection.
- Terminator. EvaluationTerminator or GenerationTerminator are compatible with this problem.

MOGA

- Problem. To test this method, [3], the Problem eva2.server.go.problems.dcu.Koduru04MultiobjectiveGrnFromMad has to be selected using the GUI. Problem parameters:
  - MOSOConverter. This parameter can be used to aggregate the different objectives into a single one. For multi objective optimisation, use *eva2.server.go.operators.moso.MOSONoConvert* .
  - Show. If set to true, the Pareto front is displayed.
- Optimiser. For this problem, eva2.server.go.strategies.MultiObjectiveEA with NSGAII archiving strategy, archive size of 50, Inserting Information Retrieval and GA optimiser (with eva2.server.go.operators.selection.SelectM selection), were used during our experiments.
- Terminator. EvaluationTerminator or GenerationTerminator are compatible with this problem.

GA+ES

- Problem. To test this method, [4], the Problem eva2.server.go.problems.dcu.Spieth05GrnFromMad has to be selected using the GUI. The final solution will display the best structure found (1 if edge exists, 0 if not) and its fitness. For the specific parameter values, please enable the outputAdditionalInfo property of EvA1 (second tab in main interface). These values are the ones displayed last for each iteration in the text file. Problem parameters:
  - Feedback. If set to true, feedback on parameter size is sent from the parameter search phase to the structure search phase. Edges having small parameter values are removed from the structure.
  - MaxConnectivity. Not used in this version.

  - ParameterSearchIterations. Number of ES iterations used during the parameter search phase (200 during the five gene experiments).
  - ParameterSearchLambda. Lambda parameter for ES (20 during the five gene experiments).
  - ParameterSearchPopSize. Initial population size for ES (25 during the five gene experiments).
  - parameterSearchMiu. Miu parameter for ES (5 during the five gene experiments).

- Optimiser. The optimiser used for this problem is GA with elitism, tournament selection (tournament size 8), and a small population, (20 individuals), due to costly evaluation.
- Terminator. During the experiments, EvaluationTerminator was used with a maximum number of evaluations set to 2,500,000. This counts fitness evaluations during parameter search (ES individuals).

GA+ANN

- Problem. To test this method, [5], the Problem eva2.server.go.problems.dcu.Keedwell05GrnFromMad has to be selected using the GUI. The final solution will display the best structure found (an array of edges that are non null) and its fitness. For the specific parameter values, please enable the outputAdditionalInfo property of EvA1 (second tab in main interface). These values are the ones displayed last for each iteration in the text file. Problem parameters:

  - ANNEpochs. The number of backpropagation epochs (20000 during the five gene experiments).
  - ANNErrorThreshold. Error threshold for the backpropagation algorithm. (1e-4 during the five gene experiments)
  - ANNLearningRate. Learning rate for backpropagation (0.1 during the five gene experiments).
  - MaxConnectivity. Maximum number of input connections allowed for each gene. (3 during the five gene experiments)
  - MaxExpressionRate. Given that we are using a sigmoid function in the neurons, the output of the ANN is restricted to (0,1). In order to allow the modelling of larger data values, we scale the data by maxExpressionRate. (1 during our experiments)
  - MaxWeightValue. Upper limit for the weights. (4 during the five gene experiments)
  - MinWeightValue. Lower limit for the weights. (-3 during the five gene experiments)

  (Parameters modelType, rateConstantsRange, kineticOrdersRange and skeletalisingThreshold are not used here, but they appear in the interface as the class for this problem is derived from a generic base class.)

- Optimiser. The optimiser used for this problem is GA with elitism, tournament selection (tournament size 4), and a small population, (25 individuals).
- Terminator.

  EvaluationTerminator was used with a maximum number of evaluations set to 2500, each evaluation consisting of running the Backpropagation learning algorithm for an ANN.

DE+AIC

- Problem. To test this method, [6], the Problem eva2.server.go.problems.dcu.NomanIba06GrnFromMad has to be selected using the GUI. Problem parameters:

  - c. This parameter controls the effect of the skeletalising term on the fitness function [6]. A value of 1000 was used during our experiments.

- FirstStageGenerations. Number of generations for the first optimisation stage.
- FirstStageRuns. Number of iterations of the first optimisation stage.
- Gene. As this methods handles each gene at a time, this parameter is used to select the gene under analysis (a value between 0 and numberOfGenes-1)
- HcLocalSearch. If set to true, Hill Climbing local search is performed on two individuals in the population each generation.
- IndividualsKeptCount. Number of individuals stored after each iteration of the first optimisation stage. These individuals will be used to initialise the population for the second optimisation stage (iterated optimisation).
- MaxIndegree. The number of incoming connections above which the individual is penalised in the evaluation of the fitness function (through the skeletalising term).
- MutationInterval. Given that the optimisation strategy is differential evolution, mutation is not performed to individuals. The mutation interval parameter defines a generation interval at which mutation in performed in the population (for diversification).

- Optimiser. Trigonometric differential evolution must be used for this method.
- Terminator. Both EvaluationTerminator and GenerationTerminator can be used. The number of evaluations/generations has to be larger than those required to complete the first stage, and define the length of the second stage.

GLSDC

- Problem. To test this method, [7], the Problem eva2.server.go.problems.dcu.Kimura03GrnFromMad has to be selected using the GUI. Problem parameters:
  - c. This parameter controls the effect of the skeletalising term on the fitness function [7]. A value of 2 was used during our experiments.
  - ConvergingPhaseIterations. Number of iterations performed during the convergence phase.
  - DifferentialThreshold. This parameter handles noise in the data during Quadratic Programming local search. It should increase for increased level of noise. Values used in our experiments were 0.001 for 0%, 0.1 for 1% and 2%, 0.15 for 5% and 0.2 for 10% noise.
  - LocalSearch. This parameter needs to be set to true.
  - LocalSearchFunctionCalls. This parameter is the maximum number of function calls allowed during Powell's local search.
  - TemplateIndividual. The template individual should have no mutation and crossover operators assigned. (eva2.server.go.operators.mutation.NoMutation, eva2.server.go.operators.crossover.NoCrossover)
- Optimiser. GA with elitism should be used for this problem, with a small population size (25 in our experiments).
- Terminator. Both EvaluationTerminator and GenerationTerminator can be used. Fitness evaluations count the number of evaluations performed during local search phase, while generations count the number of times the two phases (local search and convergence) were executed.

PEACE1

- Problem. To test this method, [8], the Problem eva2.server.go.problems.dcu.KikuchiTominaga03GrnFromMad has to be selected using the GUI. Problem parameters:
  - c. This parameter controls the effect of the skeletalising term on the fitness function [8]. A value of $1E-4$ was used during our experiments.
  - FirstStageGenerations. Number of generations for the first optimisation stage (100 in our 5 gene experiments).
  - FirstStageIterations. Number of iterations of the first optimisation stage (10 in our 5 gene experiments).
  - MaxIterationsWithNoMoreFixedParameters. Maximum number of optimisation iterations (two stages) in which no further parameters were found to be null. Optimisation stops when reaching this threshold.
  - MaxOptimisationIterations. Maximum number of optimisation iterations (two stages). This is applied only if the previous threshold is not reached.
  - SavedIndividualsCount. Number of individuals saved at the end of the first stage. These are used to initialise the population for the second stage.
  - SecondStageGenerations. Number of generations for the second optimisation stage (200 in our 5 gene experiments).
- Optimiser. GA, no elitism (important when having more optimisation iterations), tournament selection (tournament size 8).
- Terminator. Both EvaluationTerminator and GenerationTerminator can be used. The number of generations/fitness calls has to be larger than those required for one iteration (first and second stage). This threshold is used during the last optimisation iteration.

References

1. Streichert F, Ulmer H: **JavaEvA - A Java Framework for Evolutionary Algorithms**. Technical Report WSI-2005-06, Centre for Bioinformatics Tübingen, University of Tübingen 2005, [http://w210.ub.uni-tuebingen.de/dbt/volltexte/2005/1702/].

2. Tominaga D, Okamoto M, Maki Y, Watanabe S, Eguchi Y: **Nonlinear Numerical Optimization Technique Based on a Genetic Algorithm for Inverse Problems: Towards the Inference of Genetic Networks**. In *GCB99 German Conference on Bioinformatics* 1999:101–111.

3. Koduru P, Das S, Welch S, Roe JL: **Fuzzy Dominance Based Multi-objective GA-Simplex Hybrid Algorithms Applied to Gene Network Models**. In *Genetic and Evolutionary Computation - GECCO 2004* 2004:356–367.

4. Spieth C, Streichert F, Zell NSA: **Optimizing Topology and Parameters of Gene Regulatory Network Models from Time-Series Experiments**. In *Genetic and Evolutionary Computation - GECCO 2004* 2004:461–470.

5. Keedwell E, Narayanan A: **Discovering gene networks with a neural-genetic hybrid**. *Computational Biology and Bioinformatics, IEEE/ACM Transactions on* 2005, **2**(3):231–242.

6. Noman N, Iba H: **Inference of genetic networks using S-system: information criteria for model selection**. In *GECCO '06: Proceedings of the 8th annual conference on Genetic and evolutionary computation*, New York, NY, USA: ACM 2006:263–270.

7. Kimura S, Hatakeyama M, Konagaya A: **Inference of S-system models of genetic networks using a genetic local search**. *Evolutionary Computation, 2003. CEC '03. The 2003 Congress on* 2003, **1**:631–638 Vol.1.

8. Kikuchi S, Tominaga D, Arita M, Takahashi K, Tomita M: **Dynamic modeling of genetic networks using genetic algorithm and S-system**. *Bioinformatics* 2003, **19**(5):643–650, [http://bioinformatics.oxfordjournals.org/cgi/content/abstract/19/5/643].
