# Supplementary material for: Comparison of evolutionary algorithms in gene regulatory network model inference
Source: BMC Bioinformatics. 2010 Jan 27;11:59. doi: 10.1186/1471-2105-11-59 (PMC2831005; doi:10.1186/1471-2105-11-59)
Supplement: Additional file 3 — EvA2 framework. This archive contains the code and resources published by EvA2 authors (Minimum corresponding code). More details on how to use it can be found in Additional File 2. [file 1471-2105-11-59-S3.ZIP › resources/EvolutionStrategies.html]

Evolution Strategy - ES

�

# Evolution Strategy - ES

  
An ES works on a population of real valued solutions
by repeated use of evolutionary operators like reproduction,
recombination and mutation (see pseudocode in figures.
lambda offspring individuals are generated from mu parents
by recombination and mutation. After evaluating the fitness of the lambda
offspring individuals, mu individuals with the best fitness are
selected by a comma-strategy to build the parent population for the next generation.
On the other hand, a plus-strategy selects the best mu individuals
from the aggregation of parents and offspring individuals.
The properties of ES are given in the population sub frame.
