# Supplementary material for: Comparison of evolutionary algorithms in gene regulatory network model inference
Source: BMC Bioinformatics. 2010 Jan 27;11:59. doi: 10.1186/1471-2105-11-59 (PMC2831005; doi:10.1186/1471-2105-11-59)
Supplement: Additional file 3 — EvA2 framework. This archive contains the code and resources published by EvA2 authors (Minimum corresponding code). More details on how to use it can be found in Additional File 2. [file 1471-2105-11-59-S3.ZIP › resources/F1Problem.html]

f\_1 : Sphere function


# The F1 hyper-parabola function

  
The hyper-parabola function is a *n*-dimensional, axis-symmetric, continuously differentiable, convex function:

Because of its simplicity every optimization-algorithm should be able to find its global minimum at *x*=[0, 0, ... , 0]

---

More information about the F1 function can be found at:

Kenneth De Jong. *An analysis of the behaviour of a class of genetic adaptive systems.* Dissertation, University of Michigan, 1975. Diss. Abstr. Int. 36(10), 5140B, University Microflims No. 76-9381.
