# Supplementary material for: Comparison of evolutionary algorithms in gene regulatory network model inference
Source: BMC Bioinformatics. 2010 Jan 27;11:59. doi: 10.1186/1471-2105-11-59 (PMC2831005; doi:10.1186/1471-2105-11-59)
Supplement: Additional file 3 — EvA2 framework. This archive contains the code and resources published by EvA2 authors (Minimum corresponding code). More details on how to use it can be found in Additional File 2. [file 1471-2105-11-59-S3.ZIP › resources/F2Problem.html]

Generalized Rosenbrock's function


# Generalized Rosenbrock's function


This function is unimodal and continuous, but the global optimum is hard to find, because of independence through the term (*x*\_(*i*+1) - *x\_i*\**x\_i*)^2 between contiguous parameters.

Rosenbrock's function within the co-domain -5 <= *x* <= 5.

The global optimum is located in a parabolic formed valley (among the curve x^2 = x\_1^2), which has a flat ground.
  

  
The function close to its global optimum, which is: f(*x*) = f(1, 1, ... , 1) = 0.

Rosenbrock' function is not symmetric, not convex and not linear.

---

More information about Rosenbrock's function can be found at:

Kenneth De Jong. *An analysis of the behaviour of a class of genetic adaptive systems.* Dissertation, University of Michigan, 1975. Diss. Abstr. Int. 36(10), 5140B, University Microflims No. 76-9381.

Hans Paul Schwefel. *Evolution and optimum seeking.* Sixth-Generation Computer Technology Series. John Wiley & Sons, INC., 1995.

Darrell Whitley, Soraya Rana, John Dzubera, Keith E. Mathias. *Evaluating Evolutionary Algorithms. Artificial Intelligence*, 85(1-2):245-276. 1996.

Eberhard Schoeneburg, Frank Heinzmann, Sven Feddersen. *Genetische Algorithmen und Evolutionstrategien - Eine Einfuehrung in Theorie und Praxis der simulierten Evolution.* Addison-Wesley, 1994.
