# Supplementary material for: Comparison of evolutionary algorithms in gene regulatory network model inference
Source: BMC Bioinformatics. 2010 Jan 27;11:59. doi: 10.1186/1471-2105-11-59 (PMC2831005; doi:10.1186/1471-2105-11-59)
Supplement: Additional file 3 — EvA2 framework. This archive contains the code and resources published by EvA2 authors (Minimum corresponding code). More details on how to use it can be found in Additional File 2. [file 1471-2105-11-59-S3.ZIP › resources/F3Problem.html]

The step function


# The Step Function


The idea of this function is the implementation of a flat plateau (slope 0)in an underlying continuous function. It's harder for optimization algortihms to find optima because minor changes of the object variables don't affect the fitness. Therefore no conclusions about the search direction can be made.

The step function is symmetric considering the underlying function (here: f(x,y) = f(y,x)), but between the bulk constant plateau-areas not continuously differentiable.

Its minimum-area is located in the intervals: *f(x)*=*f*([-5.12,-5), ... , [-5.12,-5))=0.

---

More information about the step function can be found at:

Thomas Baeck, *Evolutionary Algorithms in Theory and Practice.* Oxford University Press, 1996.

Darrell Whitley, Soraya Rana, John Dzubera, Keith E. Mathias. *Evaluating Evolutionary Algorithms. Artificial Intelligence*, 85(1-2):245-276. 1996.

Eberhard Schoeneburg, Frank Heinzmann, Sven Feddersen. *Genetische Algorithmen und Evolutionstrategien - Eine Einfuehrung in Theorie und Praxis der simulierten Evolution.* Addison-Wesley, 1994.
