# Supplementary material for: Comparison of evolutionary algorithms in gene regulatory network model inference
Source: BMC Bioinformatics. 2010 Jan 27;11:59. doi: 10.1186/1471-2105-11-59 (PMC2831005; doi:10.1186/1471-2105-11-59)
Supplement: Additional file 3 — EvA2 framework. This archive contains the code and resources published by EvA2 authors (Minimum corresponding code). More details on how to use it can be found in Additional File 2. [file 1471-2105-11-59-S3.ZIP › resources/F5Problem.html]

Schwefel's double sum


# Schwefels double sum


Schwefel's double sum is a quadratic minimization problem. Its difficulty increases by the dimension *n* in *O(n^2)*. It is used for analysis of correlating mutations.

It possesses specific symmetrical properties:  


Its minimum is located at: *f(x)*=*f*([0, 0, ... , 0])=0

---

More information about Schwefel's double sum can be found at:

Hans Paul Schwefel. *Evolution and optimum seeking.* Sixth-Generation Computer Technology Series. John Wiley & Sons, INC., 1995.
