# Supplementary material for: Comparison of evolutionary algorithms in gene regulatory network model inference
Source: BMC Bioinformatics. 2010 Jan 27;11:59. doi: 10.1186/1471-2105-11-59 (PMC2831005; doi:10.1186/1471-2105-11-59)
Supplement: Additional file 3 — EvA2 framework. This archive contains the code and resources published by EvA2 authors (Minimum corresponding code). More details on how to use it can be found in Additional File 2. [file 1471-2105-11-59-S3.ZIP › resources/F6Problem.html]

Generalized Rastrigin's function


# Generalized Rastrigin's function


Rastrigin's function is symmetric. It is based on the simple *parabola function* (called f1 in the EvA context), but it is multimodal because a modulation term on the basis of the cosine function is added. This evokes hills and valleys which are misleading local optima.

Values used for the following illustrations: *A*=10, *ω*=2\*π, *n*=2.
  

  
Rastrigin's function within the co-domain -20>=*x*>=20

Rastrigin's function within the co-domain -5>=*x*>=5

Like Ackley's function a simple evolutionary algorithm would get stuck in a local optimum, while a broader searching algorithm would get out of the local optimum closer to the global optimum, which in this case is: f(*x*) = f(0, 0, ... , 0) = 0.

Rastrigin's function close to its optimum.

---

More information about Rastrigin's function can be found at:

Darrell Whitley, Soraya Rana, John Dzubera, Keith E. Mathias. *Evaluating Evolutionary Algorithms. Artificial Intelligence*, 85(1-2):245-276. 1996.

Eberhard Schoeneburg, Frank Heinzmann, Sven Feddersen. *Genetische Algorithmen und Evolutionstrategien - Eine Einfuehrung in Theorie und Praxis der simulierten Evolution.* Addison-Wesley, 1994.
