# Supplementary material for: Comparison of evolutionary algorithms in gene regulatory network model inference
Source: BMC Bioinformatics. 2010 Jan 27;11:59. doi: 10.1186/1471-2105-11-59 (PMC2831005; doi:10.1186/1471-2105-11-59)
Supplement: Additional file 3 — EvA2 framework. This archive contains the code and resources published by EvA2 authors (Minimum corresponding code). More details on how to use it can be found in Additional File 2. [file 1471-2105-11-59-S3.ZIP › resources/F8Problem.html]

Ackley's function


# Ackley's function


Ackley's function is multimodal and symmetrical. It is based on an exponential function and modulated by a cosine function.
The outside region is almost planar as to the growing influence of the exponential function.
In the center there is a steep hole as to the influence of the cosine function.  
Its minimum is at: *f(x)*=*f*([0, 0, ... , 0])=0.

The difficulty for an optimization algorithm is mid-graded because a simple optimization-algorithm like *hill-climbing* would get stuck in a local minimum. The optimization algorithm has to search a broader environ to overcome the local minimum and get closer to the global optima.

Ackley's function within the co-domain -20 >= *x* >= 20, *a*=20, *b*=0.2, *c*=2\*π, *n*=2.

Ackley's function close to the optimum.

---

More information about Ackley's function can be found at:

David. H. Ackley. *A connection machine for genetic hillclimbing.* Kluwer Academic Publishers, Boston, 1987.

Thomas Baeck. *Evolutionary Algorithms in Theory and Practice.* Oxford University Press, 1996.
