# Supplementary material for: Comparison of evolutionary algorithms in gene regulatory network model inference
Source: BMC Bioinformatics. 2010 Jan 27;11:59. doi: 10.1186/1471-2105-11-59 (PMC2831005; doi:10.1186/1471-2105-11-59)
Supplement: Additional file 3 — EvA2 framework. This archive contains the code and resources published by EvA2 authors (Minimum corresponding code). More details on how to use it can be found in Additional File 2. [file 1471-2105-11-59-S3.ZIP › resources/FitnessConvergenceTerminator.html]

Fitness Convergence Terminator


# Fitness Convergence Terminator

  
The fitness convergence terminator stops the optimization, when there has been hardly
any change in the best population fitness (within percentual or absolute distance) for a certain
time, given in generations or fitness calls.  
Be aware that, if the optimization is allowed to be non-monotonic, such as for (,)-ES strategies,
and if the optimum is close to zero, it may happen that the fitness fluctuates due to numeric
issues and does not easily converge in a relative measure.
