# Supplementary material for: Comparison of evolutionary algorithms in gene regulatory network model inference
Source: BMC Bioinformatics. 2010 Jan 27;11:59. doi: 10.1186/1471-2105-11-59 (PMC2831005; doi:10.1186/1471-2105-11-59)
Supplement: Additional file 3 — EvA2 framework. This archive contains the code and resources published by EvA2 authors (Minimum corresponding code). More details on how to use it can be found in Additional File 2. [file 1471-2105-11-59-S3.ZIP › resources/GO.html]

EvA2 Genetic Optimization


# The EvA2 Genetic Optimization Module

  
The Genetic Optimization module allows the application of a variety of
nature-inspired heuristics within one framework. You can combine several
datatypes as representations with specific evolutionary operators and
widely independently choose an optimization strategy. Some strategies,
however, only work with certain datatypes. Most remarkably, DE and PSO
require a real-valued representations for the moment, whereas GA, for example,
is typically run with a binary datatype but also works on real valued individuals
by just accessing the analoguous evolutionary operators.
