# Supplementary material for: Comparison of evolutionary algorithms in gene regulatory network model inference
Source: BMC Bioinformatics. 2010 Jan 27;11:59. doi: 10.1186/1471-2105-11-59 (PMC2831005; doi:10.1186/1471-2105-11-59)
Supplement: Additional file 3 — EvA2 framework. This archive contains the code and resources published by EvA2 authors (Minimum corresponding code). More details on how to use it can be found in Additional File 2. [file 1471-2105-11-59-S3.ZIP › resources/GOParameters.html]

EvA Genetic Optimization


# Genetic Optimization Parameters

  
The GO parameter class is used to change main GO optimization settings. You may:

- Choose the optimizer. Check the optimizer object for further parameters and information.
- Set post-processing parameters or leave it turned off.
- Select the problem to be optimized. Check the problem instance for further parameters and information.
- Set a random seed. For the same positive seed, an optimization run should yield the same results. Set the seed to zero to use a dynamic seed for each run (using system time).
- Define the termination criterion. Usually a maximum number of fitness evaluations is set, but
  it is also possible to choose a maximum number of generations, an absolute fitness value to be reached, a
  convergence criterion or a combination of those.

**Note:**   
The evolutionary operators used by a strategy are tightly connected to the representation used.
On the other hand, the representation is usually defined by the underlying problem, therefore,
to change the operators effecting the individuals, select the problem and set them within the
Individual class presented there. Also note, that not all optimizers can handle all types
of representations.
