# Supplementary material for: Comparison of evolutionary algorithms in gene regulatory network model inference
Source: BMC Bioinformatics. 2010 Jan 27;11:59. doi: 10.1186/1471-2105-11-59 (PMC2831005; doi:10.1186/1471-2105-11-59)
Supplement: Additional file 3 — EvA2 framework. This archive contains the code and resources published by EvA2 authors (Minimum corresponding code). More details on how to use it can be found in Additional File 2. [file 1471-2105-11-59-S3.ZIP › resources/ParticleSwarmOptimization.html]

Particle Swarm Optimization - PSO


# Particle Swarm Optimization - PSO

  
The Particle Swarm Optimization by Kennedy and Eberhardt is inspired by swarm intelligent
behaviour seen in animals like birds or ants. A swarm of particles is a set of individual agents
"flying" across the search space with individual velocity vectors. There is no selection as in
classic Evolutionary Algorithms. Instead, the individuals exchange knowledge about the space they
have come across. Each one is attracted to the best position the individual has seen so far (cognitive
component) and to the best position known by its neighbors (social component).

The neighborhood is defined by the swarm velocity, which may be a linear ordering, a grid and some others.
The influence of the velocity of the last time-step is taken into account using an inertness/
constriction parameter, which controls the convergence behaviour of the swarm.

The influence of social and cognitive attraction are weighed using the *phi* parameters. In the
constriction variant there is a dependence enforced between constriction and the phi, making sure that
the swarm converges slowly but steadily, see the publications of M.Clerc, e.g.
Typical values for the attractor weights are phi1=phi2=2.05.

The topology defines the communication structure of the swarm. In linear topology, each particle has contact
to n others in two directions, so there is a linear overlay structure. The grid topology connects a particle
in 4 directions, while the star variant is completely connected. The random variant just connects each
particle to k others by random and anew in every generation cycle.
Basically, the more connections are available, the quicker will information about good areas spread through
the swarm and lead to quicker convergence, thereby increasing the risk of converging prematurely.
By default, the random (e.g. with range=4) or grid structure (e.g. with range=2) are good choices.

The multi-swarm approach splits the main swarm in sub-swarms defined by the distance to a local "leader",
as in the dynamic multi-swarm approaches by Shi and Branke, for example. The tree structure orders the
swarm to a tree of degree k, where the fittest individuals are on top and inform all their children nodes.
In this case, the higher the degree, the quicker will information spread. HPSO is a hierarchical tree variant
by Janson and Middendorf, 2005.
