# Supplementary material for: Comparison of evolutionary algorithms in gene regulatory network model inference
Source: BMC Bioinformatics. 2010 Jan 27;11:59. doi: 10.1186/1471-2105-11-59 (PMC2831005; doi:10.1186/1471-2105-11-59)
Supplement: Additional file 3 — EvA2 framework. This archive contains the code and resources published by EvA2 authors (Minimum corresponding code). More details on how to use it can be found in Additional File 2. [file 1471-2105-11-59-S3.ZIP › resources/PhenotypeConvergenceTerminator.html]

Phenotype Convergence Terminator


# Phenotype Convergence Terminator

  
The phenotype convergence terminator stops the optimization, when there has been hardly
any change in the best population individual (within percentual or absolute distance) for a certain
time span, given in generations or fitness calls.  
Be aware that, if the optimum individual is close to zero, it may happen that its phenotype values
fluctuate due to numeric issues and do not easily converge in a relative measure.
