# Supplementary material for: Comparison of evolutionary algorithms in gene regulatory network model inference
Source: BMC Bioinformatics. 2010 Jan 27;11:59. doi: 10.1186/1471-2105-11-59 (PMC2831005; doi:10.1186/1471-2105-11-59)
Supplement: Additional file 3 — EvA2 framework. This archive contains the code and resources published by EvA2 authors (Minimum corresponding code). More details on how to use it can be found in Additional File 2. [file 1471-2105-11-59-S3.ZIP › resources/StatisticsParameterImpl.html]

Statistics Parameter Panel


# Statistics Parameter Panel

  
Statistical options are:

- Convergence rate threshold. Provided the target value is zero,
  convergence is assumed if a value smaller than this threshold is reached.
  For multi-run experiments, the number of hits is counted using this criterion.
- Number of multi-runs. To achieve statistically meaningful results on how well
  a certain optimizer works on a given problem, set this number to do several runs in a row.
  The plot will be averaged, while all data can be collected in an output file or text window.
- The fitness to plot. Define which fitness values to plot to the graph window after every
  generation. Select "best", "worst" or both.
- Result file name. If you want to collect optimization data, set this String to a desired file name.
  Optimization results will be written to the indicated file in the working directory.
- Show text output. If activated, the optimization data will also be shown in a graphical text
  window for immediate viewing.
