# Supplementary material for: Comparison of evolutionary algorithms in gene regulatory network model inference
Source: BMC Bioinformatics. 2010 Jan 27;11:59. doi: 10.1186/1471-2105-11-59 (PMC2831005; doi:10.1186/1471-2105-11-59)
Supplement: Additional file 3 — EvA2 framework. This archive contains the code and resources published by EvA2 authors (Minimum corresponding code). More details on how to use it can be found in Additional File 2. [file 1471-2105-11-59-S3.ZIP › resources/Tribes.html]

TRIBES


# TRIBES

  
TRIBES is a parameter-free PSO implementation by Maurice Clerc. It combines several adaptive
mechanisms to achieve good performance in different domains. It uses a dynamic number of particles,
starting usually with 3 and adding new ones during optimization. Therefore, the number of generations
is not directly connected to the number of fitness calls,
because the population may grow (and seldomly shrink).  
Also, there are different initialization
methods implemented which are chosen randomly when particles are created. The particles are organized
in loosely connected groups or tribes (therefore the name), creating a kind of small-world topology.
  
As TRIBES uses an error approximation to steer the adaptations, a target value should be given, so far in the first
dimension only.
